# Herbivorous Juvenile Grass Carp (*Ctenopharyngodon idella*) Fed with Genetically Modified MON 810 and DAS-59122 Maize Varieties Containing Cry Toxins: Intestinal Histological, Developmental, and Immunological Investigations

**DOI:** 10.3390/toxins14020153

**Published:** 2022-02-19

**Authors:** Gergő Gyurcsó, Béla Darvas, Ferenc Baska, László Simon, Eszter Takács, Szandra Klátyik, András Székács

**Affiliations:** 1 Agro-Environmental Research Centre, Institute of Environmental Sciences, Hungarian University of Agriculture and Life Sciences, H-1022 Budapest, Hungary; gyurcso.gergo@uni-mate.hu (G.G.); laszlosimon24@yahoo.com (L.S.); takacs.eszter84@uni-mate.hu (E.T.); klatyik.szandra@uni-mate.hu (S.K.); 2 Hungarian Society of Ecotoxicology, H-1022 Budapest, Hungary; bdarvas@bdarvas.hu; 3 Department of Exotic Animal and Wildlife Medicine, University of Veterinary Medicine Budapest, H-1071 Budapest, Hungary; baska.ferenc@univet.hu

**Keywords:** *Ctenopharyngodon idella*, *Bothriocephalus acheilognathi*, *Zea mays*, MON 810, DAS-59122, genetically modified, feeding cry toxin contained maize leaves, cry toxin measurements in grass carp, blood chemistry of grass carp, immunology of grass carp

## Abstract

Feeding experiments with juvenile grass carp (*Ctenopharyngodon idella*) fed with genetically modified maize MON 810 or DAS-59122 dried leaf biomass were carried out with 1-, 3- and 6-month exposures. Dosages of 3–7 μg/fish/day Cry1Ab or 18-55 μg/fish/day Cry34Ab1 toxin did not cause mortality. No difference occurred in body or abdominal sac weights. No differences appeared in levels of inorganic phosphate, calcium, fructosamine, bile acids, triglycerides, cholesterol, and alanine and aspartame aminotransferases. DAS-59122 did not alter blood parameters tested after 3 months of feeding. MON 810 slightly decreased serum albumin levels compared to the control, only in one group. Tapeworm (*Bothriocephalus acheilognathi*) infection changed the levels of inorganic phosphate and calcium. Cry34Ab1 toxin appeared in blood (12.6 ± 1.9 ng/mL), but not in the muscle. It was detected in *B. acheilognathi.* Cry1Ab was hardly detectable in certain samples near the limit of detection. Degradation of Cry toxins was extremely quick in the fish gastrointestinal tract. After 6 months of feeding, only mild indications in certain serum parameters were observed: MON 810 slightly increased the level of apoptotic cells in the blood and reduced the number of thrombocytes in one group; DAS-59122 mildly increased the number of granulocytes compared to the near-isogenic line.

## 1. Introduction

**Figure 1:**
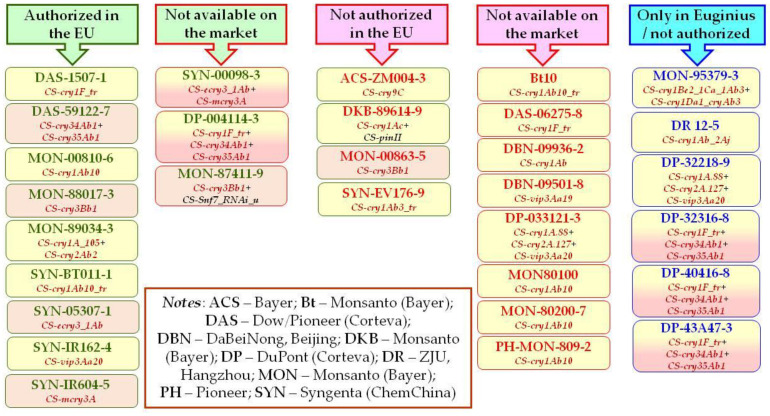
*Bt*-maize varieties containing *cry* or *vip* genes enlisted in the EUginius database of the European Union (burgundy font color; chemically modified genes—truncated, optimized, or synthetic). Comments: single genetic events authorized (green font color), not authorized (red font color), enlisted in the EUginius database (blue font color) in maize; active on Lepidoptera species (mainly *Ostrinia nubilalis*) (yellow background color), an active genetic event on Coleoptera species (mainly *Diabrotica virginifera*) (pink background color); available on the world market according to the ISAAA database (green box frame color), not available on the world market (red box frame color), current commercial status is unknown (blue box frame color).

The official database of genetically modified (GM) organisms of the European Union (EU), EUginius [1] enlisted 821 genetic events and event combinations (not just plants) in 2020, and this number rose to 856 by the end of 2021. The number of single events, from which combinations are produced, is relatively low: 307 single modifications in 13 plants are listed (2020) in the registry. Of these genetic events, 58 have reached authorizations for feed (19%) in the EU. Of the 452 GM maize varieties, 67 were single genetic event traits, and the number of genetic events in maize that received an authorization status was 19 and 30 in 2020 [2] and 2021, respectively (Figure 1).

Comparing these numbers to the corresponding ones in the database of the GM variety owners (ISAAA) [3], several contradictions are noticeable. Of 30 single genetic events in the *EUginius* database, only nine received authorization and are being distributed in the EU. Another three single genetic events are licensed in the EU but are not available on the world market. Of the licensed varieties, only MON 810 received authorization for public cultivation (sowing), but even that is severely restrained by Member State moratoria. Within the EU only Spain (~100 thousand ha) and Portugal (~4 thousand ha) grew MON 810 varieties on considerable acreage. In addition, 12 single genetic events do not have authorization in the EU, out of which only four are available on the world market; and six single genetic events without an EU license are only mentioned in the EUginius database, but their commercial status is unknown. Of these 30 single genetic events mentioned, 11 are no longer commercially available, which can be regarded as a substantial fluctuation. Compared to the ISAAA database, EUginius contains much more detailed information describing gene constructs. Thus, data in EUginius reveal that none of the *cry* and *vip* genes, the basis for insecticidal activity in GM crops in most cases, are truncated forms of the native genes in subspecies strains of *Bacillus thuringiensis*. All such plants modified by these genes are therefore transgenic, i.e., subject to authorization. The truncated protoxin genes, initially optimized for expression in plants, have been replaced by now by chemically modified (e.g., chimeric, and twin toxins) or synthetic genes. (Truncated genes are indicated in the nomenclature as _tr suffix, while chemical modification or synthesis are marked as a number tag (e.g., 0.88, 0.127 or _105) at the end of the name or a letter *e*- or *m*- prior to the gene name and after a *CS*- prefix.) In addition to the *cry* and *vip* genes, helper genes (RNAi interference resulting to down-regulation of the targeted *Snf7* gene—MON 87411; *PinII* gene produced protease inhibitor protein—DKB-89614) also appeared. On the basis of the above, our feeding experiments are related solely to genetic events MON 810 and DAS-59122, as chemical modifications of truncated native genes may be accompanied by altered biological effects.

Crystalline parasporal endotoxins of various *Bacillus thuringiensis* (*Bt*) strains, Cry toxins, have gained a significant agricultural role in recent decades [4,5]. *B. thuringiensis* strains are ubiquitous soil-borne, Gram-positive, spore-forming bacteria and insect pathogens in the environment. The identification of the composition of Cry proteins, the recognition of their unique physicochemical properties and biological specificity has led to several breakthrough results in the practice of agricultural pest control. One application is represented by the agricultural use of *Bt*-toxin-based bioinsecticides, and the other is agricultural biotechnology solutions on the basis of incorporation of gene segments encoding *Bt* toxins into GM plants. The Cry1Ab endotoxin produced by *B. thuringiensis* subsp. *kurstaki* is a protoxin with a molecular weight of 131 kDa and forming bipyramidal crystals stabilized with up to 16 disulfide bonds per molecule. It consists of three domains, a bundle of seven α-helices responsible for pore formation in the insect midgut, a set of three antiparallel β-sheets interacting with the lectin receptor resulting in toxin specificity to Lepidopteran insects, and a β-sandwich of two antiparallel β-sheets also contributing to the above two functions [6]. A transgene encoding a partially truncated form of this protoxin, a so-called preactivated toxin with a molecular weight of 91 kDa, has been introduced into maize in the genetic event MON 810. Both the protoxin and the preactivated toxin are activated in the insect organism, resulting in the reduction of its disulfide bonds and its hydrolytic cleavage to form an activated toxin with a molecular weight of approximately 63–65 kDa [5,7]. Cry34Ab1 and Cry35Ab1 are binary toxins (both required for the insecticide activity) showing specificity to Coleopteran insects. Cry34Ab1 is an endotoxin with a molecular weight of 14 kDa, not related to other *Bt* crystal proteins [6]. Similar but less significant differences are found between the microbial and plant biosynthesized forms of Cry34Ab1, as its maize-derived form is slightly truncated, one amino acid shorter at the N-terminus.

Compositional differences between the microbial and maize-derived endotoxin forms result in certain anomalies in the immunoanalytical determination of Cry1Ab. Commercial ELISA kits are generated against the bacterial protoxin (using Cry1Ab protoxin as an immunogen and as an analytical standard), and therefore they give biased detection of the preactivated Cry toxin. Immunoassay signals are calibrated to concentrations of the protoxin molecule, but antibodies directed against the protoxin are expected to have a lower affinity for the truncated preactivated toxin protein, and therefore higher concentrations of the preactivated toxin than the protoxin are required to obtain signals of the same intensity. Previously, we prepared the activated toxin by trypsin degradation of the Cry1Ab protoxin, determined its cross-reactivity with the protoxin, and found it to be between 41% and 56% [8]. This indicates that ELISA kits are suitable for the detection of both the Cry1Ab protoxin and the preactivated toxin but require a correction when detecting the preactivated toxin present in MON 810 maize, and the actual preactivated Cry1Ab toxin concentration in these *Bt* maize samples is 1.8–2.3 times higher (depending on the given commercial ELISA kit) than as detected by Cry1Ab protoxin-specific ELISA kits. This applies to the Cry1Ab values for MON 810 maize in the scientific literature, including data from the variety owner, Monsanto Corp. (currently Bayer Corp.). Due to the single amino acid difference between the microbial and plant biosynthesized forms of Cry34Ab1, no significant differences in the cross-reactivity of the microbial and the maize-derived variants to the specific antibody are expected.

We chose a freshwater herbivorous fish as the subject of our study, as it is well-known that the grass carp (*Ctenopharyngodon idella*) changes its larval predatory feeding to herbivorous behavior [9]. It is known in Asia that this fish can be foraged by farm mowed grasses or shredded corn. Our experiments were performed on 11-month young *C. idella* test animals right after diet conversion. The feeding study was preceded by a 4-month conditioning phase, during which the experimental stock was accustomed to a diet containing nearly 30% of water-swollen maize leaf shred. In the experiment started in the 11th month, Sudan grass (*Sorghum sudanense*), used as negative control, caused significant growth retardation, unlike the maize (*Zea mays*). In our study, juvenile *C. idella* individuals were certainly exposed to maximal Cry toxin treatment (dosages 3–7 μg/fish/day Cry1Ab or 18–55 μg/fish/day Cry34Ab1 toxin). Their extensive physiological exposure could further be characterized as we could also monitor the reactions of individuals that suffered a tapeworm (*Bothriocephalus acheilognathi*) infection. Such conditions could facilitate pathological entry of larger protein molecules via fish intestinal epithelial tissue atrophy occurring due to the tapeworm infection.

## 2. Results

### 2.1. Weight Measurements

#### 2.1.1. Body Weight

Weight gain of the test animals was recorded. The test animals were classified into three, small (S), medium (M) and large (L) body size groups (see Section 4.2.2), and results were evaluated accordingly. After 1 month of feeding, only the lowest body weight (group S) fed with Sudan grass (*S. sudanense*) showed significant developmental delay. Infection rates by tapeworm *B. acheilognathi* were also elevated in the M group fed with Sudan grass. The mean weight of the L group infected with *B. acheilognathi* was higher than that of the uninfected ones in the same group, suggesting edematous overcompensation. Effects of GM maize DAS-59122 or MON 810 could not be differentiated at a statistical significance from those of non-GM maize (near-isogenic varieties). No significant differences in body weight were measured in the groups fed with DAS-59122 or MON 810 compared to the near-isogenic lines even after 3 and 6 months of feeding, either.

#### 2.1.2. Abdominal Sacs

After 1 month of feeding, the S group fed with Sudan grass also remained underdeveloped according to the mass measurements of the abdominal sacs (intestines, liver, gallbladder). Even after 3 months of feeding, there were no significant differences in abdominal sac volumes between the groups fed with DAS-59122 or MON 810 compared to the near-isogenic lines. In the M body weight group, in individuals infected with tapeworm *B. acheilognathi*, there occurred a significant increase in abdominal organ weights in those fed with GM maize MON 810 compared to uninfected fish. This is related to water retention problems.

### 2.2. Hematology

#### 2.2.1. Inorganic Phosphate

Inorganic phosphate levels provide indications related to conditions of the kidney. After 1 month of feeding, 2–5 mmol/L levels were determined in the S and L groups. The results obtained did not show a difference between DAS-59121, MON 810, and the near-isogenic lines, although the levels were slightly elevated in the groups fed with Sudan grass compared to MON 810. The cause of the change was *B. acheilognathi* in both cases.

**Figure 2:**
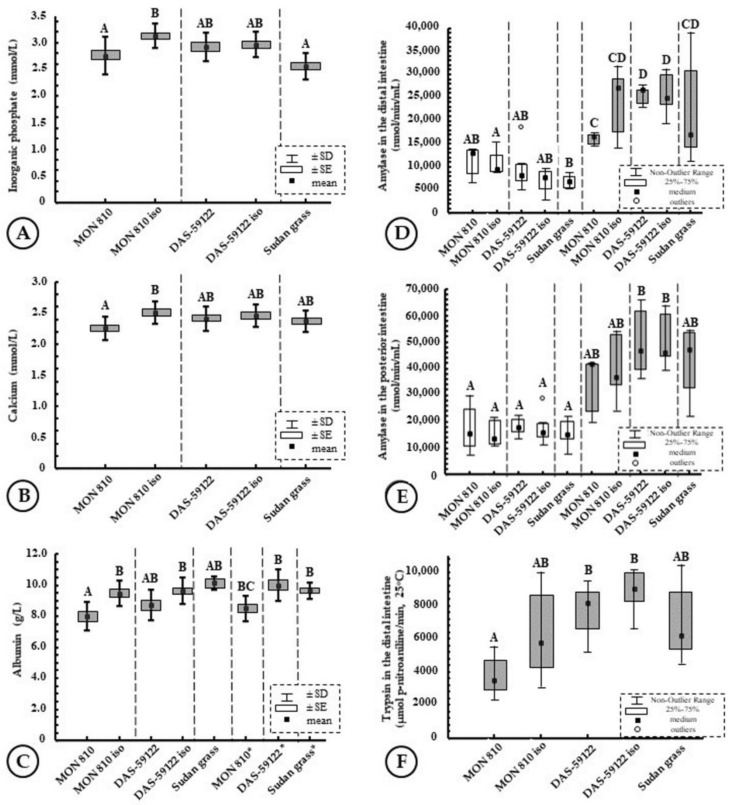
Certain parameters of the grass carp (*Ctenopharyngodon idella*) fed with GM and near-isogenic maize varieties or Sudan grass (*Sorghum sudanense*). Levels in the blood: (**A**) inorganic phosphate, (**B**) calcium, (**C**) albumin. Enzyme activities: (**D**) amylase in the distal intestine, (**E**) amylase in the posterior intestine, (**F**) trypsin in the distal intestine. Effects were separately analyzed for fish groups of medium body size (M, white boxes) and large body size (L, grey boxes), letters (A–D) above the data indicate statistically different groups at 0.05 significance level, SD: standard deviation, SE: standard error. * means *Bothriocephalus* *acheilognathi* infection.

Feeding with DAS-59122 or MON 810 for 3 months did not alter inorganic phosphate levels in the S group. In the L group, Sudan grass and MON 810 caused statistically detectable changes relative to the MON 810 near-isogenic group (Figure 2A). However, the detectable difference disappeared with the exclusion of individuals infected with *B. acheilognathi* (data not shown). DAS-59122 did not cause any discrepancy. The physiological level in the 14-month-old grass carp were 2.5–3.5 mmol/L, showing a slight decrease compared to the levels 2 months before.

#### 2.2.2. Calcium

Blood calcium levels characterize calcium metabolism. Feeding with MON 810 and DAS-59122 for 3 months did not alter calcium levels in the S group. In the L group, the level of MON 810 was significantly lower than in the near-isogenic group (Figure 2B). However, the detectable difference disappeared with the exclusion of individuals infected with *B. acheilognathi* (data not shown). DAS-59122 showed no change. The physiological level of calcium in 14-month-old grass carp was 2.0–2.7 mmol/L.

#### 2.2.3. Fructosamine

Elevated fructosamine levels indicate stress. After 1 month of feeding, fructosamine was measured in the serum in the range of 150–350 μmol/L in the S and L groups. No significant differences were found.

#### 2.2.4. Serum Albumin

The measurement is used to screen for liver and kidney diseases. After 3 months of feeding, serum albumin levels ranged 7–11 g/L. While no significant difference was detected in the S group, MON 810 significantly reduced serum albumin levels in the L group compared to the MON 810 near-isogenic group (Figure 2C). DAS-59122 showed no change. The difference in MON 810 was not due to *B. acheilognathi* but was only detectable in correlation with the body weight.

#### 2.2.5. Bile Acids

Bile acid levels provide information on the liver status. After 1 month of feeding, the occurrence of bile acids in the blood in the S group ranged 6–25 μmol/L. The large standard deviation of the data did not allow to detect the difference between GM and non-GM crops. However, infection by tapeworm *B. acheilognathi* significantly increased the serum bile acid levels of fish consuming the near-isogenic line of MON 810 (>30 μmol/L compared to an average level of 1010 μmol/L). No significant differences were detected in the L group.

#### 2.2.6. Triglycerides

This parameter is used to characterize vascular diseases affecting lipid metabolism. Treatment with MON 810 and DAS-59122 for 3 months did not alter triglyceride levels in the S and L groups. The physiological triglyceride level in 14-month-old grass carp was 1.5–3.5 mmol/L.

#### 2.2.7. Cholesterol

Cholesterol levels indicate the health status of the cardiovascular system. Feeding with MON 810 or DAS-59122 for 3 months did not alter cholesterol levels in the S and L groups. The physiological cholesterol level in 14-month-old grass carp was 3.5–5.5 mmol/L.

#### 2.2.8. Alanine Aminotransferase (ALT)

Changes in the alanine aminotransferase (ALT) levels may indicate liver disease. After 1 month of feeding, the ALT level was very low in all animals in the S and L groups and ranged 1–5 U/L. No significant differences were detected.

#### 2.2.9. Aspartate Aminotransferase (AST)

Elevated aspartate aminotransferase (AST) levels indicate tissue damage; most affected are the liver, the kidneys, and the heart. After 1 month of feeding, it ranged 40–150 U/L in the S and L groups. No significant differences were detected.

### 2.3. Enzymology

#### 2.3.1. α-Amylase Activity in the Distal and Posterior Intestine

Amylase is a digestive enzyme involved in the metabolism of complex sugars (polysaccharides). The DAS-59122 and MON 810 diets had no effect on the run of the amylase levels, unlike the body weight groups (Figure 2D–E). Enzymatic activity in the middle intestine increased significantly from 6–10 mol/L to 15–25 mol/L in the L group (Figure 2D), compared to the M group, which is likely to be attributed to faster adaptation to the herbivorous behavior.

#### 2.3.2. Trypsin Activity in the Posterior Intestine

Trypsin is a proteolytic digestive hydrolase enzyme. The DAS-59122 and MON 810 diets had no effect on the changes in its levels (Figure 2F).

#### 2.3.3. Leucine Aminopeptidase Activity in the Distal Intestine

Leucine aminopeptidase (LAP) is an enzyme that cleaves peptide bonds in proteins and is predominantly found in the liver. The DAS-59122 and MON 810 diets had no effect on the changes in its levels.

### 2.4. Cry Toxins Content in Grass Carp Tissues (ELISA)

#### 2.4.1. Determination of Matrix Effect of Grass Carp Tissues in Cry 1Ab and Cry34Ab1 Toxin Determination

**Figure 3:**
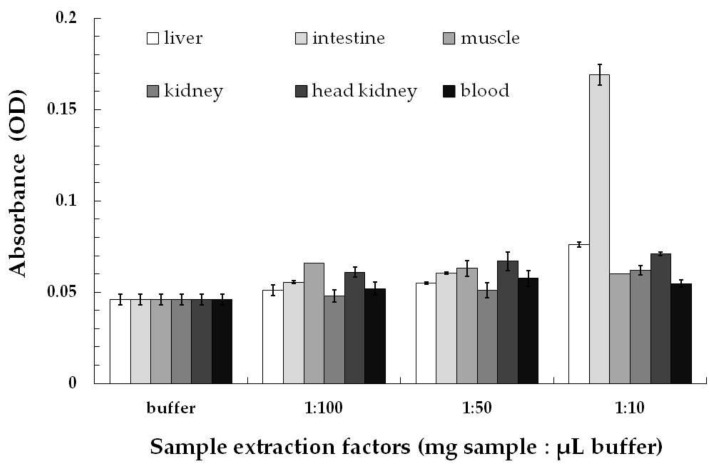
Matrix effects of grass carp tissues at different dilution rates (m/V dilution as mg sample/μL buffer) in sample preparation. Assay signals (absorbance) are expressed as optical density (OD).

Possible matrix effects in different tissues, indicated as difference ratios between assay signals expressed as optical density (OD) in the tissue extracts and in assay buffer, were determined in measurements of control samples at 1:10, 1:50 and 1:100 dilutions (mg sample/μL extraction buffer) (Figure 3). The highest matrix effect was determined for the intestine at 1:10 sample:buffer ratio, and matrix effects appeared to be diluted out, although with an apparent residual background, in all tissues analyzed at 1:50 sample : buffer ratio. Thus, a sample preparation procedure of 100 mg of tissue homogenized in 1 mL of extraction buffer was set, and extracts were measured in the ELISA systems in triplicates without any further dilution.

In recovery studies, control tissue samples at sample : extraction buffer (mg/μL) ratios of 1:10, 1:50 and 1:100 were processed and spiked at two-two levels with Cry34Ab1 or Cry1Ab each. For this purpose, 40 mg maize leaf/mL extraction buffer stock extracts were prepared from DAS-59122 or MON 810. Diluted fish tissue extracts were spiked with these maize leaf extracts at two levels (spike levels 1 and 2), 1.463 ± 0.210 and 0.731 ± 0.105 ng/mL for Cry34Ab1 and 1.030 ± 0.085 and 0.206 ± 0.017 ng/mL for Cry1Ab. Assay signals expressed as OD in spiked extracts were compared to the corresponding control signals in assay buffer, i.e., 0.287 ± 0.006 and 0.159 ± 0.009 for spike levels 1 and 2 for Cry34Ab1, respectively, and 0.265 ± 0.006 and 0.061 ± 0.002 for spike levels 1 and 2 for Cry1Ab, respectively. These spiking experiments also indicated that a dilution ratio of 1:50 of all fish tissue extract is sufficient for toxin determination at both spike levels for Cry34Ab1 and at spike level 1 for Cry1Ab (spike level 2 for Cry1Ab was overly diluted for quantitative determination of the toxin).

#### 2.4.2. Cry34Ab1 and Cry1Ab Toxin Contents Are Grass Carp Tissues

The Cry34Ab1 and Cry1Ab toxin content of ground dry maize leaf applied for the grass carp feed (GCF, see Section 4.4.2) preparation were found to be 182.9 ± 26.3 and 25.7 ± 2.1 μg/g for DAS-59122 and MON 810, respectively. There appeared no matrix effects in the determination of the toxin content in ground maize leaves and GCF under the extraction conditions (40 mg maize leaf/mL extraction buffer) by ELISA. The ratio of maize biomass in the GCF was 4.2% (m/m) dry ground maize leaf, as specified in the recipe. On the basis of the toxin content determined by ELISA, the maize biomass ratio in CGF was found to be 4.6 ± 0.7% and 4.3 ± 0.4% (m/m) for DAS-59122 and MON 810, respectively. This proved that the toxin content has not been influenced by the different steps of GCF preparation.

**Figure 4:**
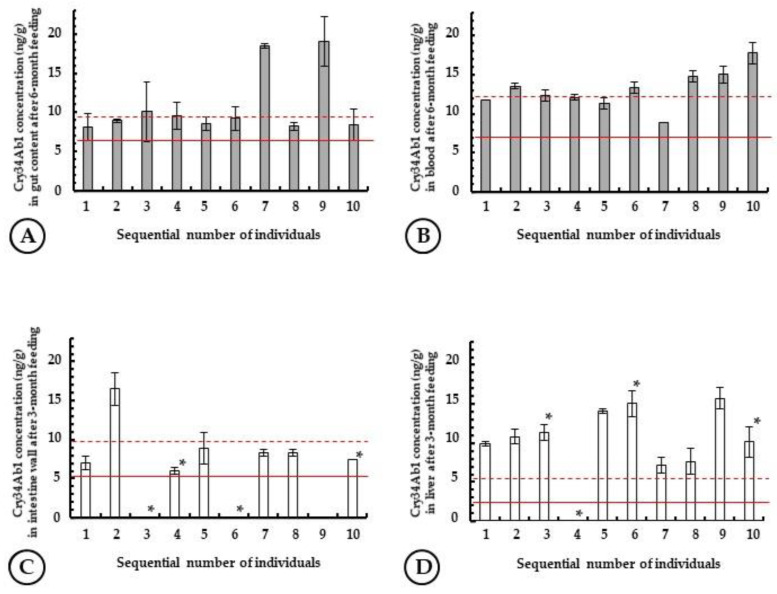
Levels of Cry34Ab1-toxin in different tissues of *Ctenopharyngodon idella* individuals after a 6-month feeding campaign: (**A**) gut content; (**B**) blood; and after 3-month feeding campaign: (**C**) intestine wall; (**D**) liver. Limit of detection (LOD) and limit of quantification (LOQ) values of given tissues are indicated with solid and dashed red lines, respectively. * indicates *Bothriocephalus acheilognathi* infection.

No measurable difference was found among individual test animals belonging to different body weight groups (M or L) in their toxin content in given tissues after the 3-month feeding period, for Cry34Ab1 or Cry1Ab. Due to the 1-day starvation stage before the sampling date, after the 3-month feeding period, for DAS-59122 the Cry34Ab1 level of the gut content (partially digested GCF) was above the limit of detection (LOD) value (6.03 ng/g) only in four samples (8.65 ± 0.62 ng/g in an average). For MON 810, the Cry1Ab level in the digested GCF decreased below the LOD of the ELISA. In a few samples, Cry1Ab concentrations slightly above the LOD were determined; however, these results do not appear to be statistically relevant. The absence of the 1-day starving stage after the 6-month feeding period resulted in 10.86 ± 4.21 ng/g Cry34Ab1 toxin concentration in the gut content, with results from all fish sampled being above the LOD value and two samples above the limit of quantification (LOQ) value (9.50 ng/g) (Figure 4A).

Although the Cry34Ab1 content of GCF rapidly decreases to 0.1–0.2% in the digestive system, the toxin appears to be detectable in blood (Figure 4B). Cry34Ab1 content of blood samples were above the LOD value (7.0 ng/mL) in all test animals with an average level of 12.59 ± 1.89 ng/mL concentration. Among grass carp sampled in the 6-month feeding study, one suffered from tapeworm infection, and the Cry34Ab1 concentration in the blood of this fish was 17.77 ± 1.40 ng/mL, which may indicate accelerated toxin penetration through the intestine wall damaged (atrophy) by the tapeworm (*B. acheilognathi*) infection.

The intestine tissue is a very complex matrix in Cry34Ab1 determination, and toxin concentrations determined by ELISA were below the LOD value (5.26 ng/g) in eight cases, moreover the highest SD value among individuals was determined for the intestine, among the tissues studied. The Cry34Ab1 content was 8.90 ± 3.50 ng/g and 7.32 ± 2.24 ng/g for the 3- and 6-month feeding studies, respectively (Figure 4C). For liver samples, the toxin content of one sample was below the LOD (2.39 ng/g), the average Cry34Ab1 concentration was determined to be 11.31 ± 3.07 ng/g) (Figure 4D).

For muscle, kidney, and head kidney (*pronephros*) samples, 4.97 ± 1.56 ng/g (LOD: 3.25 ng/g), 9.76 ± 2.26 ng/g (LOD: 4.96 ng/g) and 5.52 ± 1.43 ng/g (LOD: 2.39 ng/g) Cry34Ab1 toxin concentrations were determined, respectively. Tapeworm infection was 46.7 ± 5.8% in DAS-59122 treatment in the 3-month feeding study, there was no difference among grass carp groups of different body weight. Although *B. acheilognathi* infection appears to possibly facilitate elevated occurrence of Cry34Ab1 in the blood of the fish (see above), such correlation has not been seen in other tissues investigated. Samples of the tapeworm showed Cry34Ab1 toxin content of 7.13 ± 0.40 ng/g, but there occurred no negative correlation between the toxin concentration in the tapeworm and in the gut content of the fish.

### 2.5. Histology

**Figure 5:**
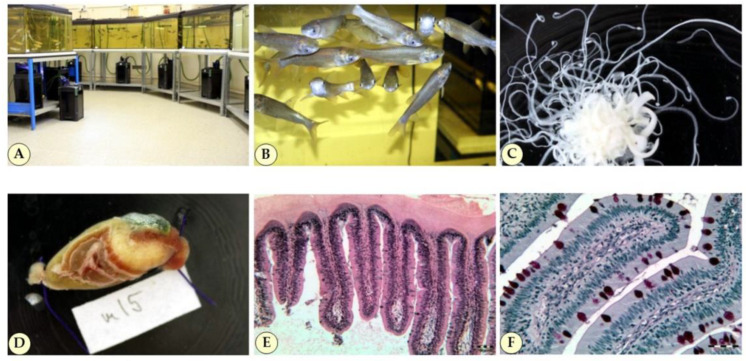
(**A**) Rearing *Ctenopharyngodon idella* in the experiment; (**B**) 1-year-old *C. idella*; (**C**) tapeworm *Bothriocephalus acheilognathi* isolated from the test animals; (**D**) abdominal bag; (**E**) distal intestine; (**F**) goblet cells (Photo: (**A–D**)—Darvas, B.; (**E**,**F**)—Baska, F.).

Histological examinations were carried out using tissues of *C. idella* individuals in the feeding experiments (Figure 5). Histology indicated no abnormalities in the liver. Similarly, no significant differences were seen in the gastrointestinal tract in the size of the crypts in the distal and posterior intestine (average crypt depth of 180–200 μm and epithelial cell height of 16.16 μm). The number of goblet cells in the same field of view was around 80–90 and 50–60 after 3 and 6 months of feeding, respectively. None of the GM maize varieties (DAS-59199, MON 810) caused additional changes in the intestines for the two dominant cell types (Figure 5E,F) other than from developmental causes. The effect of infection by *B. acheilognathi* (Figure 5C) was well observed in the intestine, and the intestinal mucosa became thinner: instead of the physiological 380–460 μm, it showed a thickness of only 35–60 μm. In infected individuals, goblet cells proliferated. The hepatopancreas showed abnormalities (lipid infiltration, fibrosis).

### 2.6. Apoptotic Cells (TUNEL Assay)

**Figure 6:**
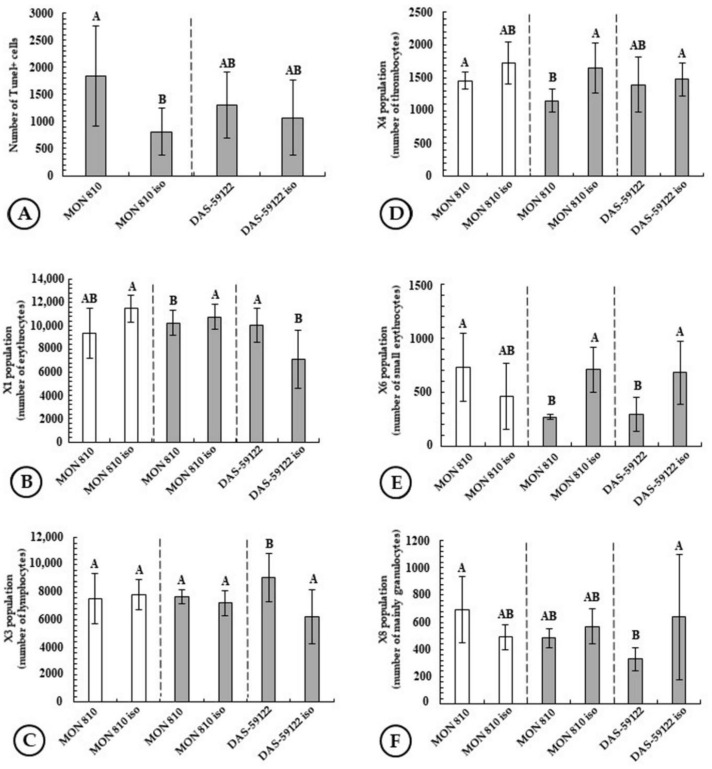
The effects of 6-month feeding: the numbers of (**A**) TUNEL+ cells; (**B**) *Erythrocytes*; (**C**) *Lymphocytes*; (**D**) *Thrombocytes*; (**E**) the X6 fraction (small *Erythrocytes*); (**F**) *Granulocytes*.

DNA strand breaks are often related to programmed cell death: apoptotic cells can induce DNA breaks, and DNA damage triggers apoptosis. Apoptosis induced DNA breaks can occur mediated by the immune system that, when certain cells are identified, sends a signal that causes the hereditary material to be sliced up. In our experiments, 6-month feeding with a diet containing DAS-59122 did not cause a DNA damaging effect detectable by the TUNEL assay (the number of apoptotic cells in the L-weight group was 1310 ± 607, while that in the near-isogenic control line was 1081 ± 684), whereas feeding with a diet containing MON 810 resulted in a significant increase in the number of apoptotic cells in the blood (1849 ± 924), relative to its isogenic line (813 ± 427) (Figure 6A).

### 2.7. Immunology

#### 2.7.1. Erythrocytes

Red blood cells in fish are nucleated, although these nuclei are inactive, and the role or involvement of red blood cells as mediators in the immune response of fish is not yet explored sufficiently. Characteristically, leukocyte–antigen interactions take place in fish in the kidney, head kidney, spleen, gills and intestine; therefore, these organs also serve as immunological sites in the immune response in fish. In our experiments, 6-month feeding with a diet containing MON 810 maize had no observed effect neither in the L, nor in the S body weight groups. In contrast, feeding the test animals with a diet containing DAS-59122 resulted in a significant increase in the number of adult erythrocytes in the blood (10040 ± 1414) compared to that seen in animals fed with its near-isogenic line (7083 ± 2455). Incidentally, the latter negative control did not differ significantly from the values obtained with MON 810 and its near-isogenic line (Figure 6B).

The X6 cell population isolated in flow cytometry proved to be a set of erythrocytes smaller in size than the majority of the cells. This number decreased in L body weight groups for both DAS-59122 and MON 810. Such a decrease in the X6 cell population has not been seen in the M body weight group fed with a diet containing MON 810 (Figure 6E).

#### 2.7.2. Lymphocytes

Lymphocytes are one of the bases of immune defense in fish. Within the 6-month feeding test in our study, DAS-59122 significantly increased the number of lymphocytes (9169 ± 1771) in the L body weight group compared to its near-isogenic line (6267 ± 1972). The latter negative control did not differ significantly from the values obtained with MON 810 and its near-isogenic line (Figure 6C).

#### 2.7.3. Thrombocytes

Thrombocytes in fish, as analogues of platelets in mammals, are elements responsible for blood clotting. In the feeding experiment, 6-month feeding with a diet containing MON 810 significantly reduced the number of thrombocytes in the blood (1162 ± 177) compared to its near-isogenic line (1653 ± 396) in the L body weight group. However, this effect was not detectable in the M body weight group consuming MON 810 (Figure 6D).

#### 2.7.4. Granulocytes

Various forms of the granulocytes are elements of immune defense in fish that also possess certain granulocytes possibly related to neutrophils in mammals. In this study, 6-month feeding with a diet containing DAS-59122 significantly reduced the number of granulocytes (332 ± 82) compared to its near-isogenic line (644 ± 466) in the L body weight group at DAS-59122. This value remained below that of MON 810 (488 ± 64) and its near-isogenic counterpart (576 ± 131) (Figure 6F).

## 3. Discussion

A research letter in The Lancet in 1999 [10,11] initiated a rather critical nutritional and foraging revision of first-generation (transgenic) GM crops variety groups in Europe. The article reported that an experimental GM potato variety carrying a *Galanthus nivalis* lectin transgene caused gastrointestinal disturbances, which the authors assessed primarily by weight measurement (the developmental order of certain organs was altered) and histological studies in rats, and the plant vector used for transformation was also suspected as a causative agent. The leader of this experiment triggering a great deal of controversy at the turn of the millennium, Árpád Pusztai and his colleagues did not observe a similar effect in relation to another experimental transgenic pea variety (containing an α-amylase inhibitor transgene) and found that GM variety suitable for feeding purposes [12]. None of these experimental events appeared later in the market as commercial varieties, but the implications of the experiments on GM potatoes, although the majority of the results of which have never been published in scientific articles, greatly influenced the assessment of subsequent feeding studies.

Variety owner companies disputed the alleged general impacts. Commercial and widespread feeding of MON 810 maize to pigs has not been associated with developmental abnormalities or histological problems [13,14,15,16]. In experiments with DAS-59122 maize in Sprague-Dawley rats, DuPont researchers have not recorded any effects on body parameters [weight/gain, food consumption/efficiency, clinical signs of toxicity, mortality, ophthalmology, neurobehavioral (functional observational battery and motor activity) assays, clinical pathology (hematology, clinical chemistry, coagulation, and urinalysis), and pathology (organ weights and gross and microscopic pathology)] [17]. De Vendômois et al. [18] have reported different effects for Cry1 (MON 810) and Cry3 toxins (MON 863) for each genetic event: “Our analysis clearly reveals for the 3 GMOs new side effects linked with GM maize consumption, which were sex- and often dose-dependent. Effects were mostly associated with the kidney and liver, the dietary detoxifying organs, although different between the 3 GMOs. Other effects were also noticed in the heart, adrenal glands, spleen and hematopoietic system. We conclude that these data highlight signs of hepatorenal toxicity, possibly due to the new pesticides specific to each GM corn. In addition, unintended direct or indirect metabolic consequences of the genetic modification cannot be excluded.” The debate continues to this day. The retracted (and re-published) report by Séralini et al. [19,20] concerning a group of glyphosate-tolerant cultivars (MON 603) has been particularly divisive. In the nearly twenty-year feeding history of GM crops, no proof of harmlessness or harmfulness beyond reasonable doubt has been reached, although economic damages have certainly not been evidenced based on studies on mammalian species [21,22].

The results of studies on fish have been reported to a much lesser extent, but with sufficient rigor [23]. Hemre et al. [24] reported an experiment with juvenile (smolt, 104 ± 15 g) Atlantic salmon (*Salmo salar*) fed for 3 months with normal fish feed, RoundupReady (RR) soybean feed (RR, glyphosate tolerant) or unmodified soybean feed. No mortality occurred during feeding of MON-04032-6 soybeans, and no significant difference was measured in the growth of the different groups. Spleen weights were significantly higher than those in the group fed with normal soy but were similar to the group on the control diet. The weight of the distal intestine was significantly reduced in the soy-fed groups. Among the mean number of erythrocytes within blood parameters initially decreased, being the lowest in the group fed with GM soy by the half of the period studied, no significant difference remained by the end of the experiment. Bakke-McKellep et al. [25] worked with juvenile animals (smolt, 104 ± 15 g). The fish were fed for 3 months with a standard fish diet, and diets containing RR soy (MON-04032-6) or unmodified soy. It renders it difficult to determine that soy feed caused inflammation in the intestines of the salmon, but the inflammation in the distal intestine was more extensive in the group fed RR soy, than in the group of animals fed with unmodified soy. Histological examinations revealed that vacuoles instead of enterocytes appeared in supranuclear cells in the groups fed with soybean, compared to the group fed with normal fish diet, and the width of the *lamina propria* (connective tissue layer of the intestinal mucosa) increased in the group fed with RR soy. RR soy increased the activity of lysozyme and alkaline phosphatase enzymes in the head kidney. In addition, soy diets reduced leucine aminopeptidase and maltase activity in the distal intestine. Based on the study, it was concluded that RR soy can be safely used.

Sagstad et al. [26] carried out a 28-day study with Atlantic salmon (*S. salar*) (average weight 707 ± 131 g) with diets containing 150 and 300 g/kg of RR soy. The protein and n-6 fatty acid content decreased, while the n-3/n-6 fatty acid ratio increased in the muscle significantly with increasing GM soy content in the feed. Moreover, decreased triglyceride and glucose levels were measured in the blood plasma. Spleen weight was higher in the group fed with a diet containing GM soy compared to the group fed with normal soy, and hemoglobin levels were negatively correlated with spleen size, suggesting a weak immune response.

Sissener et al. [27] performed a chronic study with RR soy in Atlantic salmon (*S. salar*), in a 7-month experiment during the parr–smolt transformation from a freshwater habit to a marine habit (initial average weight 40 g). The diets contained 25% of GM and 26% of normal soy. Significant differences were found in body weights: fish in the group fed with GM soybean were larger, but this difference disappeared towards the end of the experiment, and no significant differences appeared in organ masses. The distal intestine was significantly larger in the group fed with unmodified soy, and significantly higher triacylglycerol levels were detected in the blood [26]. Other parameters varied independently of the feed, depending on the level of development.

Unlike experiments with GM soy, which involve a range of histologically characteristic gastrointestinal inflammations due to soybean itself, feeding experiments with GM maize are much more related to our study. The effects of MON 810 maize at levels of 15% and 30% of maize kernels in the diet on juvenile Atlantic salmon (post-smolt) were assessed in an 82-day feeding study [28]. Body weights were found to be significantly lower in the group fed GM maize compared to the group fed with unmodified maize, but none of the groups fed with maize differed significantly from the control group fed with normal fish feed. Food intake differed significantly, being lower in the group fed with GM maize. Liver weight/body weight ratios were significantly higher in the group fed with a higher proportion of maize in the diet. Distal intestine weight/body weight ratios were also significantly higher but did not differ from the group fed with normal fish feed [28].

In our study, no differences were found in body or abdominal sac weight of the grass carp (*C. idella*) fed with GM maize, regardless of the feeding duration (1-, 3- or 6-month). However, the two fish species studied, Atlantic salmon and grass carp, differ significantly. Maize leaves may have been more suitable for the digestion system of the herbivorous grass carp we studied, than the starch-rich corn kernel for the predatory salmon.

Sagstad et al. [29] reported that superoxide dismutase-1 (SOD) activity was higher in the liver and the intestine, while catalase activity was lower in the liver than in the control. Stress protein (HSP70) levels were also higher in the liver. Among white blood cells, the proportion of granulocytes and monocytes was higher, but the proportion of lymphocytes was lower in the group fed with a diet containing MON 810. In our study of feeding *C. idella* for 6 months with GM maize, neither an increase in the number of granulocytes, nor a decrease in the number of lymphocytes were observed (Figure 4C,F). In the study of Gu et al. [30,31], diets compared contained ~20% of maize (MON 810 and unmodified) or 16.7% soy. Young salmon individuals were found to be more tolerant of the soy diet, with no inflammation found in the intestines. In contrast, GM maize induced minor but significant differences in digestive enzyme levels. Decreased intestinal activity of leucine aminopeptidase and maltase, as well as decreased levels of intestinal bile salts were observed, while amylase activity increased. In our study, no alterations were detected in leucine aminopeptidase levels relative to the control after 3- and 6-month feeding of *C. idella*. Regarding amylase activity, we found differences only between body weight groups (Figure 2D,E)

Previous studies have demonstrated that the quantity of Cry1Ab protein that survives intestinal digestion is very low and have also confirmed that the Cry1Ab protein is absent from the blood and organs of livestock fed with *Bt* maize for extended periods [13,14,15,16,32,33,34,35]. In our study, we also proved that concentration of Cry1Ab and Cry34Ab1 toxins decrease to a very low level in the gastrointestine system due to digestion or decomposition. For Cry1Ab toxin, this level was under/at the LOD values of the ELISA system applied in the analytical measurements. For eliminating or decreasing the matrix effects of different tissues, 1:50 dilution with extraction buffer was applied in the sample preparation step. Since the Cry1Ab concentration was very low in the intestine system, the presence of the toxin slightly above the LOD in given tissue samples was proven only in few individuals by ELISA assay, in other ones it was below the LOD. Our results for Cry1Ab toxin determination by ELISA method are similar to the study of Buzoianu et al. [16], where long-term and trans-generational effects of MON 810 maize (PR34N44, Pioneer Hi-Bred) were investigated in pigs, during gestation and lactation, on maternal and offspring immunity. Cry1Ab toxin was detected neither in the serum of the sows nor in the plasma, heart, kidney, spleen, muscle, or brain of the offspring. Matrix effects of sow tissues were explored by Takács et al. [36]. In addition to studies on feeding swine with MON 810 maize, similar assessments have been carried out on the Atlantic salmon (*S. salar*) as well [30,31], in which Cry1Ab toxin could not be detected in tissues of the fish statistically significantly above the LOQ. Nonetheless, an apparent occurrence of the toxin near the LOD was observed [37], just as in our present study.

Unlike Cry1Ab, the Cry34Ab1 toxin was detected in grass carp whole blood samples above the LOD value in our study, moreover it was detectable in all tissues (although not in all individuals) investigated. Due to residual matrix effects at up to 1:50 sample dilutions and variation among individuals, the average Cry34Ab1 concentration determined in grass carp tissue samples were above but still close to the LOD value. For these reasons, subtle effects may not be evident unless multiple health indicators are examined.

As seen, the effects of feeding MON 810 to the herbivorous *C. idella* in our study differ from those obtained with the predatory *S. salar*. The latter experiments were performed using maize kernels, while ours were carried out with dried leaves, containing approximately 20 times higher Cry1Ab toxin content than the kernels [8,38,39]. The results of our study indicated a slight induction of stress and immune response. Feeding DAS-59122 or MON 810 *Bt* maize confirmed very mild significant effects in *C. idella* in given but not all body weight groups [40,41,42]. In our opinion, the feeding effects of different genetic events cannot be generally deemed harmless or hazardous but require a case-by-case assessment. The use of the fresh, pre-emergence vegetative mass of densely sown MON 810 or DAS-59122, when applied in the feed mixed with other plants, appears to be suitable for *C. idella*. However, widespread use of GM feed in fish would require further, mainly immunological testing, but such biological tests do not yet play key roles in the authorization of plant protection products, either.

## 4. Materials and Methods

### 4.1. Maize Varieties

#### 4.1.1. MON 810

The DK-440 BTY GM maize variety of DeKalb Genetics Corp. belonging to the MON 810 variety group (YieldGard^TM^, MaizeGard^TM^) developed by Monsanto Corp. (Monsanto → Bayer) was grown under an official experimental cultivation approval. The near-isogenic parental line applied as a control was DK-440. Seeds were obtained from Monsanto Hungária Kft. The MON-00810-6 variety was originally produced by the biolistic particle delivery (gene gun) procedure. According to the EUginius database [1], a functional gene *CS-cry1Ab10* has been introduced into the maize genome. The truncated version of the *cry1Ab* gene of *B. thuringiensis* pathovar. *kurstaki* encodes a preactivated Cry1Ab toxin protein with a molecular mass of 91 kDa (816 amino acids). As a result, the GM maize is primarily resistant to the larvae of the European corn borer (*Ostrinia nubilalis*), but also affects the larvae of the cotton bollworm (*Helicoverpa armigera*) in Europe [43]. The ISAAA database [3] currently (unlike in its previous versions) surprisingly enlists four types of transgenes (genes from foreign species): *cry1Ab1* (=*CS-cry1Ab10*), *goxv247* (from *Ochrobactrum anthropi* strain of LBAA), *cp4 epsps* (from *Agrobacterium tumefaciens* strain CP4), *nptII* (derived from *Escherichia coli* Tn5 transposon), the first of which being responsible for the production of Lepidoptera-specific Cry1Ab toxin [43], the second and third being selection markers for glyphosate tolerance, and the fourth being a marker gene. The contradiction regarding the occurrence of these four transgenes in a single-trait (non-stacked) genetic event is rather confusing.

MON 810 and its near-isogenic line were grown on a calcareous loam soil semi-field (location: Herman Ottó út, Budapest, Hungary) that received only organic manure and no pesticide treatments. Drip irrigation was applied between June and August.

#### 4.1.2. DAS-59122

The Herculex^TH^ RW GM maize variety of Pioneer Hi-Bred International, Inc. belonging to the DAS-59122-7 variety group developed by Dow and DuPont (now known as Corteva in conjunction with Pioneer) was grown under an official experimental cultivation approval. The seed was obtained by the Hungarian Ministry of Environment and Water. The DAS-59122 variety was originally produced by an *Agrobacterium tumefaciens*-mediated plant transformation method. According to the *EUginius* database [1], functional genes *CS-cry34Ab1* (encoding a Cry34Ab1 toxin protein with a molecular mass of 14 kDa), *CS-cry35Ab1* (encoding a Cry35Ab1 toxin protein with a molecular mass of 44 kDa) and *CS-pat* (encoding phosphinothricin N-acetyltransferase from *Streptomyces viridochromogenes*) have been introduced into the maize genome. The *cry* genes used herein, active against Coleoptera larvae *(i.e*., ineffective on adults) are truncated versions of the genes of the nonmotile *B. thuringiensis* strain PS149B1. The *CS-pat* gene is responsible for glufosinate tolerance. The binary toxins Cry34Ab1 and Cry35Ab1 render the maize roots resistant to the larvae of the western corn rootworm (*Diabrotica virginifera*). The ISAAA database [3] has the same content for DAS-59122.

Cultivation of DAS-59122 and its near-isogenic line was performed as described above (see Section 4.1.1).

### 4.2. Ctenopharyngodon Idella

#### 4.2.1. Pre-Conditioning

Fish used in the experiments were obtained from a pond farm (Aranykárász Bt., Szarvas, Hungary) cooperating with the Research Institute for Fisheries and Aquaculture (HAKI) of the National Agricultural Research Centre, Hungary. Fish were transferred into the aquarium system of HAKI at 5 months of age, above an individual size of ~20 g (the typical stage of conversion of the feeding habit) and were delivered to our laboratory at the age of 7 months. A 3-month preliminary feeding mainly with a diet containing *Spirulina* algae was conducted, during which no mortality occurred, and then the individuals were selected into three groups by body weight. The 10-month-old offspring were subjected to a 1-month maize-diet habituation performed with ground and wetted DK-440 maize leaf biomass. Foraging *C. idella* with maize leaves is characteristic mainly to Vietnam and Southeast Asia [44,45].

#### 4.2.2. Separation by Body Weight

All treatments were performed in 3 groups of fish of similar size in parallel in a well-tempered (20–22 °C) common room with an 18:6 light: dark photoperiod. For this purpose, the fish at the age of 11 months, after slight narcotization (see Section 4.6.1), were separated into the following groups: 25–26 g/individual (S group), 27–28 g/individual (M group), and 31–32 g/individual (L group).

### 4.3. Bothriocephalus acheilognathi

It was revealed during dissection of 1-month old fish examined that nearly half of the animals were infected with the Asian fish tapeworm *B. acheilognathi* from the rearing site (Figure 5C). *B. acheilognathi* infection rates correlated neither with the body weight of the fish individuals, nor with the food consumed. On average, 4–10 live tapeworms were found in the infected animals. Physiological parameters tested were statistically evaluated first for the entire (tapeworm-free and -infected) population tested, and later, when possible, for the two subpopulations separately, unless the number of replicates were insufficient for statistical analysis (due to low number of individuals and/or accidental sample loss) for tapeworm-infected individuals. No mortality was observed during the short term (1- and 3-month long) feeding tests, and the longer (6-month) test was performed only with the tapeworm-free M and L groups. As all fish individuals were labeled with unique identifiers in the study, infected individuals could be evaluated separately.

### 4.4. Feeds

#### 4.4.1. Ground Vegetable Biomass

The maize leaf biomass used in the study was collected at the beginning of maize germination (R1 phase) and contained an order of magnitude more toxin (182.9 ± 26.3 and 25.7 ± 2.1 μg/g Cry34Ab1 or Cry1Ab, respectively) than in the kernels [8,38,39] commonly used for foraging. The leaves were dried at room temperature in the shade, were ground (avoiding overheating of the mill), and were then stored in airtight containers at 4 °C until use. The Cry toxin content of the leaf biomass was measured by ELISA for each feed preparation (see Section 4.9).

#### 4.4.2. Wet Feed—Grass Carp Feed (GCF)

The composition (component ratios specified as m/m% in the final preparation) of the wet feed material was prepared in a modified version of the method by Du et al. [46,47]: 4.2% dried and ground maize leaf biomass was swollen in 27% distilled water at 30 °C for 2–3 h at room temperature, with stirring in every 10 min. When even the leaf veins lost their white color, 4.2% of casein, 0.1% of Jolovit vitamine solution (containing: 14,000,000 IU/L vitamine A, 1.4 g/L vitamine B1, 2.1 g/L vitamine B2, 1.4 g/L vitamine B6, 0.014 g/L vitamine B12, 1,400,000 IU/L vitamine D3, 10,500 IU/L vitamine E, 0.035 g/L biotin, 0.175 g/L vitamine K3, 14 g/L nicotinamide, 0.175 g/L folic acid, 140 g/L choline chloride, 6.55 g/L D-panthenol and 0.01 g/L β-carotene), 0.2% of ascorbic acid, and 1% of frozen red mosquito biomass (sliced to 3 mm pieces) were added, and the biomass was mixed. In a separate vessel, 1.5% of gelatine and 3.8% of corn starch were added to 58% of cold distilled water, was allowed to swell during 10 min of stirring, and the suspension was brought to boiling with continuous stirring over low heat. After 5 min of boiling while avoiding burning of the mass on the walls of the vessel, the final suspension cooled down to 30 °C in a cold-water bath. The cooled gelatine-starch suspension was then poured onto the leaf biomass with continuous stirring, the mixture was stirred until homogeneity (5 min), was filled into freezing trays, allowed to jellify at 5–15 °C, and was frozen at −20 °C.

Feeding with the ground corn leaf diet prepared resulted in only very slow weight gain on the long run (0.5–2 g/3 months). Feeding with Sudan grass resulted in weight loss in the **S** body weight group (3–4 g/3 months), while in weight gain, although with a very large variance, in the L group.

### 4.5. Experimental Setup

#### 4.5.1. Setup of the Aquaria

Excessively filtered, 200 L aquaria (Wiwal, Uciechów, Poland) and 1500 L/hr external filters (Aqua Nova NCF 1500, Jablonowo-Pomorskie, Poland), as well as an electromagnetic air compressor with oilless lubrication and stone air atomizers (Hailea, ACO-318, Raoping, China) without plastic fixtures immersed in the water were used (Figure 5A). Ventilated tap water with 20% water change per week, and 18:6 h light:dark scattered light avoiding overexposure were used. Due to the sufficient efficiency of bottom cleaning, no sand or gravel was used at the bottom of the aquarium.

#### 4.5.2. Feeding Treatments

Twenty fish were placed in each aquarium (Figure 5B), and the number of test animals continuously decreased with the removal of individual fish for sampling. Animals processed for experimental purposes were labeled with unique identification numbers, weighed, sampled for tail blood, dissected, after which the abdominal sac was weighed. This was followed by collection and fixation of histological specimens, detection of *B. ancheilognathi* infection, and collection of tissue and *B. ancheilognathi* samples for Cry toxin measurement. Treatments were carried out with GCF diets nearly 30% of which were of plant origin, containing MON 810 GM maize, MON 810 near-isogenic maize, DAS-59122 GM maize, DAS-59122 near-isogenic maize or Sudan grass, with feeding provided every day between 8 and 9 AM at the time of changing illumination from dimmed to direct light. All feed has been consumed by the test animals within 1 h. This unavoidably resulted in a certain variability in individual feed consumption, but feed residues in the aquarium had to be avoided to maintain proper water quality.

#### 4.5.3. One-Month Feeding Treatments (Acute Effects)

Feeding was carried out with 1.6 g/fish/day GCF, with 1.7 ± 0.1 µg/g Cry1Ab and 13.5 ± 1.9 μg/g Cry34Ab1 detected in GCF, resulting in dosages of 2.8 ± 0.2 μg Cry1Ab and 21.5 ± 3.1 μg Cry34Ab1 toxin/fish/day in the feed with MON 810 and DAS-59122, respectively. Tests carried out: inorganic phosphate, fructosamine, bile acids, ALT, AST (see Section 4.7.1, Section 4.7.3, Section 4.7.5, Section 4.7.8, Section 4.7.9).

#### 4.5.4. Three-Month Feeding Treatments (Sub Chronic Effects)

Feeding was carried out with 2.0 g/fish/day GCF, with 2.2 ± 0.2 µg/g Cry1Ab and 16.8 ± 2.4 μg/g Cry34Ab1 detected in GCF, resulting in dosages of 4.4 ± 0.4 μg Cry1Ab and 33.6 ± 4.8 μg Cry34Ab1 toxin/fish/day in the feed with MON 810 and DAS-59122, respectively. Tests carried out: inorganic phosphate, calcium, serum albumin, triglyceride, cholesterol, α-amylase, trypsin, leucine aminopeptidase (see Section 4.7.1, Section 4.7.2, Section 4.7.4, Section 4.7.6, Section 4.7.7, Section 4.8.1, Section 4.8.2, Section 4.8.3).

#### 4.5.5. Six-Month Feeding Treatments (Chronic Effects)

Only tapeworm-free individuals in the M and L body weight groups were subjected to blood sampling and dissection after the 6-month feeding experiments. Dehelmintization was carried out with anthelmintic drug praziquantel at a dosage of 15 mg/kg (the S body weight group did not survive this treatment). All fish individuals were processed for the tests. Feeding was carried out with 2.4 g/fish/day GCF, with 2.7 ± 0.2 μg/g Cry1Ab and 20.2 ± 2.9 μg/g Cry34Ab1 detected in GCF, resulting in dosages of 6.4 ± 0.5 µg Cry1Ab and 48.4 ± 7.0 μg Cry34Ab1 toxin/fish/day in the feed with MON 810 and DAS-59122, respectively. Tests carried out: inorganic phosphate, fructosamine, TUNEL assay, immunology tests (see Section 4.7.1, Section 4.7.2, Section 4.12, Section 4.13).

### 4.6. Body Weight Measurement and Dissection

At the end of the feeding period (after 1, 3 or 6 months) test animals were labeled with unique identifier numbers and processed for testing. Feeding was no longer administered the day before the measurement.

#### 4.6.1. Sleep Induction

Prior to body weight measurement and dissection, test animals were subjected to sleep induction. Ingredients of the fish-specific sleep inducer used were 40 g benzocaine (Sigma E1501, Sigma Aldrich, Saint Louis, MO, USA), 90 mL 10% hydrochloric acid, 160 mL 96% ethyl alcohol, 1000 mL distilled water, 10 mL Tonogen (1 mg adrenaline/epinephrine/mL water) in injectable solution (Richter Gedeon, Budapest, Hungary). A 1–2 mL of fish sleep inducer was put into 100–200 mL of dechlorinated tap water. Each fish was put into the sleep inducer solution and kept there until it became immobile (usually about 10–20 s).

#### 4.6.2. Body Weight Measurement

After sleep induction and soaking up the moisture from the fish body surface, the entire body weight of each test animal was measured individually.

#### 4.6.3. Abdomen Sack Organs

In addition to total body weight, the weight of the abdominal sacs dissected from animals was also measured (Figure 5D). This usually resulted in less variance than that of the total body weights.

### 4.7. Hematological Tests

The caudal fin of the grass carp was cut off at the beginning of the anal fin using a scalpel. After removing the caudal fin, the fish (wound downwards) was placed into a 5 mL Falcon tube containing 2 mL of phosphate buffer saline (PBS) with heparin (100 IU/mL), and a drop of grass carp blood was added to the content of the Falcon tube. Thereafter, the contents of the tubes were quickly suspended using a 1 mL pipette, and subsequently, the Falcon tube was closed and put-on ice. Hematology measurements were carried out using an Olympus AU400 Chemistry Analyzer (Olympus Co., Tokyo, Japan).

#### 4.7.1. Inorganic Phosphate

The level of inorganic phosphate was measured using a Beckman Coulter kit (No: OSR 6122, Beckman Coulter Ireland, Maryfort, Ireland) and following the manufacturer’s protocol at 37 °C in a measurement range of 5–179 mg/L. The instrument automatically printed out the measured data in mg/dL units, which were converted to mmol/L.

#### 4.7.2. Calcium

The level of calcium was measured using a Dialab kit (No: DO1376, Wiener Neudorf, Austria) and following the manufacturer’s protocol in a measurement range of 0.01–5 mmol/L.

#### 4.7.3. Fructosamine

The level of fructosamine was measured using a Dialab kit (No: 301140, Wiener Neudorf, Austria) and following the manufacturer’s protocol in a measurement range of 10–1000 μmol/L.

#### 4.7.4. Serum Albumin

The level of serum albumin was measured using a Beckman Coulter kit (No: OSR 6102, Beckman Coulter Ireland, Maryfort, Ireland) and following the manufacturer’s protocol at 37 °C with a sensitivity of 0.15 g/L. The instrument automatically printed out the measured data in mg/dL units, which were converted to g/L.

#### 4.7.5. Bile Acids

The level of bile acids was measured using a Diasys kit (12238 99.10930, Diasys DiaSys Diagnostic Systems GmbH, Holzheim, Germany) and following the manufacturer’s protocol in the range of 2–200 μmol/L.

#### 4.7.6. Triglycerides

The level of triglycerides was measured using a Beckman Coulter kit (No: OSR 6118, Beckman Coulter Ireland, Maryfort, Ireland) and following the manufacturer’s protocol at 37 °C in the range of 0.14–9.39 g/L. The instrument automatically printed out the measured data in mg/dL units, which were converted to mmol/L.

#### 4.7.7. Cholesterol

The level of cholesterol was measured using a Beckman Coulter kit (No: OSR 6116, Beckman Coulter Ireland, Maryfort, Ireland) and following the manufacturer’s protocol at 37 °C in the range of 0.33–4.88 g/L. The instrument automatically printed out the measured data in mg/dL units, which were converted to mmol/L.

#### 4.7.8. Alanine Aminotransferase (ALT)

The level of ALT was measured using a Beckman Coulter kit (No: OSR 6107, Beckman Coulter Ireland, Maryfort, Ireland) and following the manufacturer’s protocol in the range of 3–386 U/L.

#### 4.7.9. Aspartate Aminotransferase (AST)

The level of AST was measured using a Beckman Coulter kit (No: OSR 6109, Beckman Coulter Ireland, Maryfort, Ireland) and following the manufacturer’s protocol in the range of 3–433 U/L.

### 4.8. Enzyme Assays in Distal and Posterior Intestine Samples

#### 4.8.1. α-Amylase

The level of α-amylase activity was detected by colorimetry using the Amylase Activity Assay kit (No: ab102523; Abcam, Cambridge, UK) and following the manufacturer’s protocol with a sensitivity of 0.2 mU/well.

#### 4.8.2. Trypsin

The level of trypsin activity was detected by colorimetry using the Trypsin Activity Assay kit (No: ab102531; Abcam, Cambridge, UK) and following the manufacturer’s protocol in the range of 10–100 mU/well.

#### 4.8.3. Leucine Aminopeptidase (LAP)

The level of leucine aminopeptidase activity was detected using the LAP ELISA kit (No: MBS029262); MyBiosource, San Diego, CA, USA) and following the manufacturer’s protocol in the range of 0.25–10 mmol/L.

### 4.9. Cry Toxin Determination by Enzyme Linked Immunosorbent Assay (ELISA) in Plant and Animal Tissues

Cry1Ab and Cry34Ab1 toxin content in ground dry maize leaves applied in preparation of GCF, in GCF and in certain tissues of grass carp sampled after 3- and 6-month feeding studies was determined using the Abraxis Bt-Cry1Ab/Ac ELISA kit (Abraxis Inc., Warminster, PA, USA) and Bt-Cry34AB1 quantitative complete ELISA kit (CD Bioscience Inc., Shirley, NY, USA) and following the manufacturers’ protocols. For ground dry maize and GCF 40 mg of samples were homogenized in 1 mL of manufacturer-provided extraction buffer and then diluted by 1:1000 and 1:250, respectively. Immunoassays were carried out in 96-well ELISA microplates. In the colorimetric assay, after adding the stop solution the color development (OD) was determined at 450 nm wavelength using an iEMS microtiter plate reader (Labsystems, Helsinki, Finland). For analytical measurements, grass carp of the M and L body weight groups were sampled after 3 months (10–10 fish/group), and of the L body weight group (6 fish/group) was sampled after 6 months of feeding. After 3 months, digested GCF from intestine, empty intestine and liver tissues were sampled. In the 6-month feeding study, the sampling procedure was completed by kidney, head kidney, muscle, and blood samples. As an additional measurement after 3 months, we determined the Cry toxin content in *B. acheliognathi*, where the tapeworm infection occurred. To avoid cross-pollution of grass carp tissues by the Cry toxin contained GCF in the intestine system, the posterior and anterior ends of the abdominal bag were tied up by a surgical thread. During the dissection of the abdominal bag, separation of the intestine was the last step. After removal of digested GCF, the intestine was cleaned 3 times with physiological salt solution. Samples were collected in Eppendorf tubes and were immediately sunk in liquid nitrogen to stop enzymatic reactions. Samples were stored at −20 °C until immunoanalytical measurement.

The two commercial ELISA assays applied in toxin determination have been developed for quantitative determination in maize kernel and leaf, thus the first step of analytical application was to optimize the immunoanalytical method for digested GCF and fish organs. Thus, we determined the optimal sample : buffer ratio in the sample extraction process for minimalization of possible matrix effects and determined the LOD and LOQ values for each tissue on the basis of matrix calibration obtained with control samples.

### 4.10. Histology Tests

#### 4.10.1. Tissue Fixation and Slice Preparation

Tissue samples from the S, M and L body weight groups were fixated in an aqueous formaldehyde solution made isotonic with a 0.8% sodium chloride solution in water. After fixation for at least 1 day, samples were dehydrated by a sequential treatment with ethanol solutions of gradually increasing concentrations, clarified by impregnation with xylene, embedded in Paraplast, and 4–5 μm slices were cut by a microtome.

#### 4.10.2. Staining for Microscopy

Histological staining of hematoxylin-eosin and periodic acid-Schiff (PAS) stain [48] were applied (Figure 5E). The height (from the base membrane to the brush border) of cylindrical epithelial cells, as well the depth of glandular crypts from the distal and posterior intestine sections were measured at 12 points on slices from fish not infected with tapeworm *B. ancheilognathi*. In addition, the number of mucus-producing goblet cells was also counted in the distal and posterior intestine (Figure 5F).

### 4.11. TUNEL Assay

For TUNEL staining the APO-BrdU TUNEL Assay Kit (No: A23210; Thermo Fisher Scientific, Waltham, MA, USA) was used following the manufacturer’s protocol. The samples were measured by flow cytometry using a FACSAria III cell sorter (Becton Dickinson, San Jose, CA, USA).

### 4.12. Immunological Tests

#### 4.12.1. DiOC6 Staining for Cytometry

To each 200 μL of blood sample suspension, 800 μL of PBS was added. The cells were suspended, centrifuged at 1500 rpm for 5 min. The supernatant was removed, and the pellet was resuspended in 1 mL of PBS. Each cell suspension was stained with DiOC6(3) at 50 nM for 15 min in the dark. The staining procedure was stopped by adding 2 mL of PBS to each sample. Subsequently, the samples were centrifuged at 1500 rpm for 5 min, the supernatant was removed, and the pellet resuspended in 1 mL of PBS. The last procedure was repeated three times. The samples (1 mL of suspension each) were then placed on ice and transported in a dark box, to protect it from the light [49,50].

#### 4.12.2. Cytometry of DiOC6-Stained and TUNEL-Stained Whole Blood Preparation Samples

Flow cytometric analysis was performed on a FACSAria III cell shorter Becton Dickinson, San Jose, CA, USA), using the FACSDiva Software 6.0. Blood cell sizes and densities were assessed using forward and side-angle scatters. The green fluorescence of the DiOC6 was detected in the FITC channel between 500–550 nm. In the case of the DiOC6-stained samples, 30,000 cells were analyzed from each sample (each grass carp). In the case of the green, fluorescent TUNEL-stained samples, 10,000 cells were analyzed from each sample (each grass carp).

#### 4.12.3. Morphology-Based Cell Identification

Flow cytometric analysis was performed on a FACSAria III cell shorter (Becton Dickinson, San Jose, CA, USA), using FACSDiva Software 6.0. Blood cell sizes and densities were assessed using forward and side-angle scatters. The green, fluorescence of the DiOC6 was detected in the FITC channel between 500–550 nm. The FSC-FITC dot plots were used to identify populations to be sorted and subjected to morphological identification. The identified populations were compared across various treatment groups. In total, 100,000 cells were collected from each population. Each population was centrifuged at 1500 rpm, the pellets resuspended in 100 μL of PBS, pipetted onto glass slides and subsequently, heated (70 °C) and air dried. The smears were fixed in methanol for 2–3 min, stained with Giemsa fluid for 8–10 min, washed, dried, and covered. The stained smears were observed and photographed under a light microscope equipped with a video camera linked to a computer. The blood cells of the various populations were identified and counted in 30 fields of vision using a 40X lens. The various treatment groups of grass carps were compared based on the characterized cell populations.

### 4.13. Statistical Analysis of Data

Data were evaluated with ANOVA, Tukey or Spjotvoll–Stoline tests using software Statistica (StatSoft Inc., Tulsa, OK, USA, and TIBCO Software Inc., Palo Alto, CA, USA).

## Data Availability

Not applicable.
